# Quittor

**Published:** 1899-11

**Authors:** L. A. Merillat

**Affiliations:** Secretary of M’killip Veterinary College, Chicago


### DEPARTMENT OF SURGERY.

By L. A. Merillat, V.S.,
SECRETARY OF M'KILLIP VETERINARY COLLEGE, CHICAGO.

ASSISTED BY

W. E. A. Wyman, M.D.V., V.S., MILWAUKEE, WIS.

J. A. Sloan, B.Sc., M.D.V., SAN FRANCISCO, CAL.

Dr. Robert Jay, M.D.V., WEST LIBERTY, IA.

### Quittor.

*Definition.* A quittor is a chronic, purulent, inflammatory disease located on the lateral aspect of the foot of the horse, and characterized by lameness, suppuration, necrosis, and the formation of fistulæ. It usually attacks the lateral cartilages, but other structures, including the os pedis and coffin-joint, may be involved in the morbid process.

*Etiology.* They result from an infective inflammation emanating from an abrasion, either at the sole, wall, or coronet. Nail-pricks and corns of the sole, cracks of the wall, and wounds or disease of the integument in the region of the coronet are the more common causes, but any lesion that will permit the entrance of pyogenic organisms to the region of the lateral cartilages may prove a cause of quittor. The severity of a quittor does not, however, depend upon the severity of the primary lesion, but upon the virulence and progress of the infection.

*Classification.* As in all quittors the lateral cartilages sooner or later become involved, and in 99 per cent. of all cases are the cause of perpetuating the morbid process, the universal designation "cartilaginous quittor" is justifiable; but since those serious fistulous conditions of the tendons and coffin-joint, as well as minor superficial fistulæ of the coronet, which either abort spontaneously or yield promptly to simple treatment, are usually placed in the same category, the following classification is necessary: First, cartilaginous quittor—the typical quittor of the horse; second, cutaneous quittor, which results from treads, cuts, or scratches, and never progresses beyond the superficial soft structures, and, third, tendinous quittor—a more or less acute condition involving the perforans tendon, navicular sheath, or even the second interdigital articulation.

*Course.* The course is essentially chronic, regardless of the immediate cause. The successive formation of abscesses which discharge and leave fistulæ do not usually heal spontaneously, but close only to appear in the adjoining tissues. With rational surgical interference and rigid antiseptic after-treatment the process may be limited to from fifteen to thirty days; but without interference the course is indefinite, often continuing for years. Fatalities may occur from septicæmia, synovitis, or decubitus, due to laminitis of the opposite foot.

*Symptoms.* More or less severe lameness with painful tumefaction in the region of the lateral cartilage are the first signs of a forming quittor, and when both these symptoms are present the diagnosis is certain. The lameness of quittor is that of any foot-injury in the heel. In advancing the foot is placed carefully to the ground to avoid concussion. The posterior stride is shortened to avoid the weight. In severe cases the heels do not reach the ground. Abscesses soon form within the tumefaction and discharge their purulent contents through one or more openings at the coronet. The discharge, while quite persistent, may be slight or very profuse, varying greatly from time to time. The tumefaction increases as the process continues until the foot presents an unsightly, distorted appearance. The openings heal, to reappear at other points, until the enlargement is studded with cicatrices.

*Prognosis.* The prognosis is usually favorable, although considerable time may be required to effect a cure; but when the process has involved the navicular sheath, joint, or the os pedis, death or chronic lameness may result.

*The Treatment of Cartilaginous Quittor.* With antiseptic treatment of traumata, corns, etc., the disease may be prevented; but as soon as the formation of a quittor is evident no amount of external antiseptic treatment will cut short or cure the condition, and surgical treatment alone will effect a prompt cure. The old method of cauterizing a fistulæ is successful in only a very small percentage of cases, as well as being a slow, tedious procedure. The treatment must be surgical, and the surgeon must be mindful of the condition at hand—*i. e.*, he must recognize the fact that a favorable termination cannot be expected until the insulting agent which is the cause of keeping up the diseased condition—the diseased lateral cartilages—must first be removed. To accomplish this a number of methods have been recommended. Prof. Beyer has advocated a method which is familiar to all readers of veterinary literature, consisting of exposing, then removing, the diseased portions of the cartilages by making a flap of the laminæ, coronet, and skin which covers them. The trouble with this procedure has been that a primary union of the flap is absolutely necessary to ensure good results.

The method adopted at the McKillip clinic consists of first classifying all cartilaginous quittors according to cause into two kinds: First, those caused by lesions above the coronet, and, second, those caused by lesions below the coronet—*i. e.*, from corns, nail-pricks, quarter-cracks, etc. As the aim of the surgical treatment is simply to remove the diseased section of the lateral cartilages, it is readily seen that this discrimination must be made, because in the former the upper part of the cartilage is the seat of the trouble, while in the latter the base is mainly involved. The seat of the primary lesion can usually be ascertained from the history; but in the absence of a good history, careful paring of the heel will reveal evidence of a pre-existing lesion, even in very old quittors emanating therefrom.

The animal is always cast and secured, and the diseased foot, after thorough disinfection, is rendered bloodless with an Esmarch bandage or by other suitable means. If caused by a lesion above the coronet the cartilage is exposed by making a deep incision about four inches long, bringing into view its entire upper border. As much of the cartilage as is desired is then removed with a sharp curette. Packing with antiseptic oakum and the application of an occlusive bandage completes the operation. If the cause has been a nail-prick or corn the base of the cartilage is exposed by removing a crescent-shaped section of hoof along the entire length of the tumefaction and as low as the base of the os pedis. The upper border of the cartilage is exposed by the incision described in the first operation. This brings the whole cartilage into view with the exception of the part covered by the coronary band. The necrotic area is readily recognized by its greenish color, and often sequestri of considerable size are found to exist. These, together with all of the diseased portion of the cartilage, are then removed and the part dressed as in the first operation. The coronary band need never be severed, as all parts of the cartilages can be reached from above and below it.

The after-treatment of these operations is important, and if properly executed the cure should be certain and prompt. If the procedure has been an aseptic one, and the occlusive bandage perfectly applied, the dressings may remain unmolested for several days. When the dressings are removed the wound is washed and subjected to a prolonged syringing with a 1 : 1000 mercuric chloride solution, and then the antiseptic oakum and bandage reapplied, to again remain as long as is consistent with the existing conditions.

J. A. Sloan, B.S, M.D.V.

---

### Surgical Items.

In treating septic wounds, and especially in obstetrical operations, all wounds of the hands should be protected against infection. By dipping the hands into dilute acetic acid, or vinegar, the smarting sensation will reveal the presence and location of the minutest abrasion which would otherwise be overlooked. Small wounds can be safely protected by painting them with tincture of chloride of iron; larger ones should in addition be bandaged, or, if possible, covered with a rubber nipple.—L. A. M.

Slight rises of temperature after an operation, even in cases which give no septic results, warn the surgeon that he has probably erred in some way, and that his methods and material require pretty thorough overhauling.—*International Journal of Surgery.*

